# Re: A prototype non-invasive urodynamic test to estimate voiding reserve in normal adult males. By Shafik Shoukry, Mostafa Elmissiry, Ahmed Abulfotooh, Ahmed Moussa, Wally Mahfouz, Waleed Dawood, Aly Abdel-Karim and Mohamed Hassouna

**DOI:** 10.1080/2090598X.2019.1642620

**Published:** 2019-09-17

**Authors:** Fawzy F. Farag

**Affiliations:** Urology, Sohag University Hospital, Sohag, Egypt, fawzy.f.farag@gmail.com

Voiding LUTS can affect up to 60% of elderly men [1] and they can be so severe that surgical intervention for bladder outlet de-obstruction is required in up to 25% of men aged >60 years [2]. The current standard diagnostic test to exclude bladder BOO secondary to benign prostatic enlargement (BPE) is pressure–flow studies [3]. It involves the insertion of a pressure sensor catheter in the urinary bladder to measure the intravesical pressure (P_ves_), and another catheter in the rectum to measure the intra-abdominal pressure (P_abd_). Ultimately, the actual detrusor muscle pressure (P_det_) is calculated as the outcome of the equation: P_det_ = P_ves_ – P_abd_ [4]. However, pressure–flow studies are invasive in nature and can be associated with some morbidity, including urethral discomfort, pain, haematuria, and UTI. Therefore, several non-invasive approaches have been investigated for the diagnosis of lower urinary tract dysfunction including: penile-cuff test during voiding [5], real-time monitoring of detrusor muscle strain during voiding [6], and near infrared spectroscopy to monitor bladder wall haemodynamic changes during voiding [7].

In the Shoukry et al. [8] study, the authors aimed to identify what they called ‘voiding reserve’ in a group of healthy men after excluding urodynamic BOO in them. To do so, they underwent flow tests daily for 6 days using a condom catheter connected to rigid test tubes of varying graded lengths.

Of interest, although the pressure–flow study was carried out as mentioned in the methods section, the outcome of the test was not reported in the manuscript. Having carried out urodynamic studies a day before the test, it would have been of value to measure the detrusor reserve for these men as described by Yalla et al. [9], i.e. the P_det_ at maximum urinary flow (Q_max_) (isovolumetric pressure).

Some points need to be considered on reading this interesting manuscript. First, the arbitrary proposal of threshold values to define ‘normal’ flow in the study group and the arbitrary proposed height of the test tubes. Second, physical and physiological properties of the urethra and bladder neck are different from a rigid restful tube and therefore results need to be interpreted cautiously. Last, one important component of the equation is still missing in this method, which is the actual P_det_ contributing to the voiding reserve because the calculated voiding reserve in the current work is actually the outcome of both detrusor pressure and intra-abdominal pressure; the authors might consider finding a way to measure this in further studies. I look forward to seeing the further application of the current findings in adult men with LUTS/BPE to validate its clinical significance as a potential non-invasive diagnostic tool for BOO.
